# Characteristics of Young People Reporting a Low Sexual Desire in Switzerland

**DOI:** 10.1007/s12119-023-10171-2

**Published:** 2023-11-20

**Authors:** Lorraine Chok, Joan-Carles Suris, Yara Barrense-Dias

**Affiliations:** Research Group on Adolescent Health, Department of Epidemiology and Health Systems, Center for Primary Care and Public Health (Unisanté), University of Lausanne, Lausanne, Switzerland

**Keywords:** Gender, Health promotion, Prevention, Sexual behaviours, Youth

## Abstract

This study explores the characteristics of young female and male adults (mean age 26.3 years) reporting a low sexual desire. A 2017 Swiss national survey was carried out among young adults. Participants were divided into two groups based on their level of sexual desire: Low and High. Overall, 17.2% of females and 5.7% of males reported a low sexual desire. At the multivariate level, compared to females in the High group, females in the Low group had higher odds of being dissatisfied with their social life and with their sexual life in the past 4 weeks, having no current relationship and having accepted several times sexual intercourse without really wanting. Compared to males in the High group, males in the Low group had higher odds of reporting a non-heterosexual attraction (trend), having no current relationship and having accepted several times sexual intercourse without really wanting. The prevailing idea that young people, particularly males, always have high levels of sexual desire may not be accurate and warrants further consideration. These results show that sexual desire encompasses social aspects and underscore importance of addressing how stereotypes and social norms may influence our sexuality.

## Introduction

In 2018, *The Atlantic* (Julian, 2018) raised the issue of a decline in sexual activity and desire among young people. The phenomenon, described as ‘The Sex Recession’, seems to indicate that youths nowadays would start their sex life later and would have less sexual activity than previous generations despite what public opinion might think. The article was based on various results such as the ones found by the Centers for Disease Control and Prevention’s Youth Risk Behavior Survey (CDC, 2021) according to which the percentage of 14–18-year-olds who had ever had sex dropped from 54.1% to 38.4% between 1991 and 2019. This trend had already been raised in another study (Twenge et al., 2017) with 20–24-year-old participants born in the 1980s and 1990s (the “Millenials” and “iGen” generations) being less sexually active compared to the previous generation (“GenX”).

According to the Sexual Desire Inventory (SDI), sexual desire is broadly defined as the interest in sexual activities (Dawson & Chivers, 2014). Therefore, one of the reasons that could explain the decline of sexual activity among young people could be a decrease in sexual desire. Sexual desire is a part of the definition of sexuality according to the World Health Organization (2010) and a broad concept that cannot be reduced to genital sexual behaviors or intercourse (Hinderliter, 2013). Moreover, a low sexual desire does not equate to a Hypoactive Sexual Desire Disorder (HSDD) which is defined in the DSM-IV as ‘persistently or recurrently deficient (or absent) sexual fantasies and desire for sexual activity, which causes personal distress’ (Hinderliter, 2013). Contrary to a HSDD, a low sexual desire may be temporary and/or not problematic for the individual who experiences it. Finally, neither HSDD nor a low sexual desire should be assimilated to asexuality which is not a disorder but can be defined as a normal variation of human sexuality. Furthermore, someone can have a low sexual desire without being asexual (Hinderliter, 2013).

The level of sexual desire may be related to a variety of factors such as socio-demographic ones (Ammar et al., 2014), health status (WHO, 2010), body image (Dosch et al., 2016a), adolescence sexuality (Boislard et al., 2016), relationship status (Dawson & Chivers, 2014), level of sexual satisfaction (Dosch et al., 2016b), sexual practices such as masturbation (Krejčová et al., 2017) and history of lifetime unwanted sexual experiences (Maseroli et al., 2018; Turchik & Hassija, 2014). Therefore, sexual desire is influenced by contextual, physiological and relationship factors which may affect females and males in different ways (Dawson & Chivers, 2014). Although the lack of sexual desire has been found to be more prevalent among females than males (Dawson & Chivers, 2014), males can experience a low or inexistent sexual desire as well (Nimbi et al., 2020a). However, in addition to the fact that sexual desire in youths and young adults is little discussed (Boislard et al., 2016), the lack of sexual desire is often studied from a female perspective (Nimbi et al., 2020a). As reported in a German study (Beutel et al., 2018), when addressed, males’ lack of sexual desire is centered on aging populations. Therefore, to date, there is a gap in scientific literature on sexual desire among young people on the one side and among males on the other. The purpose of this study is to determine the characteristics of both female and male young adults reporting a low sexual desire.

## Materials and Methods

Data were obtained from a national survey on sexual health and behaviors of young people in Switzerland carried out in 2017 (Barrense-Dias et al., 2018). The initial sample was provided by the Swiss Federal Office of Statistics and was representative of the 24–26-year-old population on 30 September 2016 living in Switzerland in terms of sex, language (French, Italian or German) and canton of residence. This age range ensured that most participants would be sexually active and, at the same time, sufficiently young to be able to remember their sexual life course. However, due to the delay in putting online the questionnaire (between June and November 2017 instead of January 2017) the participants were between 24.8 and 28.1 years old with a mean age of 26.3 when completing the survey. Selected individuals were invited to participate in the study through an information letter sent by postal mail, which included a link to an online anonymous questionnaire. The final sample included 7142 participants (response rate 15.1%). To correct a slight over-representation of females from the French-speaking part of Switzerland, analyses were weighted by sex and canton of residence. Weights were computed for all respondents having completed the questionnaire entirely. Final weights range between 0.25 and 2.74, but 90% of the weights are comprised between 0.65 and 1.51. A few number of individuals indicated “other” as sex and the weight for these people was fixed to one as we do not have reliable and official statistics at national level. Ethics clearance in agreement with Swiss law was given by the Ethics committee in research of the canton.^1^ A detailed description of the survey method can be found elsewhere (Barrense-Dias et al., 2018).

### Variables

#### Dependent Variables

Sexual desire was assessed through the question from the Sexual desire and the female sexual function index (FSFI) (Isidori et al., 2010): “*In the last 4 weeks, how would you rate your level of sexual desire or interest*”? with 6 possible answers dichotomised into Low (very low or none at all, low) and High (moderate, high, very high). A total of 5,124 (48.9% females; mean age 26.3 at the time of survey) answered the question. Given the fact that the sample of people who did not recognise themselves as males or females was too small (0.012%) and that sexuality-related issues may be different for this population, this article focuses on those who responded as either males or females.

#### Independent Variables

Socio-demographic characteristics included birthplace (Switzerland/other), place of residence (urban/rural), living with parents (Yes/No), linguistic region (French/German/Italian), financial and social life satisfaction, professional situation (work/studies/other: housewife/househusband, unemployment, disability insurance, social assistance, job search, other), attained education level (tertiary/below) and perceived puberty onset compared to their peers.

We measured the social life and financial satisfaction of participants by asking them how satisfied they were on a scale of 1 (very dissatisfied) to 10 (very satisfied) (Anderson, et al., 2012).

Their perceived self-reported onset of puberty (Berg-Kelly & Erdes, 1997) was assessed through the question “*If you think about the age at which you started your puberty, compared with other same-age and gender youths, would you say that you were*” with five possible answers ranging from “very much in advance” to “very much later”, trichotomised into “advanced”, “on time”, and “delayed” (Tremblay & Frigon, 2005).

The Low and High groups were also compared on their affective and sexual life with questions on sexual attraction, lifetime use of pornography, number of lifetime sexual partners, current relationship status and sexual life satisfaction in the last 4 weeks. Sexual attraction was assessed through the following question: “*What best describes how you feel?”* and dichotomised the respondents between “exclusively heterosexual” (only attracted to people of the opposite sex/strongly attracted to people of the opposite sex) and “non-exclusively heterosexual” (attracted to both women and men/only attracted to people of the same sex/strongly attracted to people of the same sex/gender has no impact/attraction for nobody/I do not know/I am not sure). We measured the participants’ pornography use through the following question: “*Have you ever surfed on a website to see pornographic movies or images?*” with three possible answers: no, never/yes, once/yes, several times. The question was taken from the IFOP survey (IFOP CAM4, 2013). Given the fact that we were interested in the regularity aspect of watching pornography, we dichotomised the answers into “no or once”, and “yes, several times”. We asked the participants their number of lifetime sexual partners and classified them into four categories: none, one, two or three, four or more. The categories for sexual partners were based on previous studies (Baumann et al., 2011; Eaton et al., 2010) and on the distribution of the responses. We assessed the participants’ current relationship status and grouped the answers into three categories: having no partner (“none”), having one or more casual partners or having both a steady partner and casual partners (“casual”), and having a steady partner (“steady”). Current sexual satisfaction was measured by asking the question “*In the last 4 weeks, how satisfied were you with your sex life in general?*” and the answers were dichotomised into unsatisfied (“moderetely/very unsatisfied”) and other (“neither satisfied nor dissatisfied or moderately/very satisfied”).

We assessed three types of undesired sexual experiences: having ever accepted sexual intercourse without really wanting (never, once, several times), unwanted sexual intercourse or contact (never, once, several times with the same/various persons) and sexual assault/abuse (yes/no). Having ever accepted sexual intercourse without really wanting may be categorized in the sexual compliance group (Auderset et al., 2021) (i.e. the act of consenting to unwanted sexual activity (Darden et al., 2019)). Unwanted sexual intercourses include many situations that some people may situate between consensual sex and sexual assault such as emotional or verbal pressure, sexual intimidation, dating violence, and so on (Akre et al., 2013). The question on sexual abuse was taken from SMASH02 survey (Narring, et al., 2002) and defined as “a sexual assault or abuse occurs when a family member or someone else touches where you would not want to be touched, or when someone does something to you about your sexuality but he or she should not do it”.

We assessed the participants’ health through several items. First, we asked them how they perceived their general health status (excellent/very good, good, poor/very poor). Then, to assess general mental health (depression and anxiety), we used the mental health inventory (MHI-5), a brief questionnaire with 5 items referring to the last 4 weeks (Berwick et al., 1991); for instance, “*How much of the time during the past 4 weeks have you… (1) been a very nervous person? Or (2) felt so down in the dumps that nothing could cheer you up*?” Score ranges from 0 to 100 with the higher the score, the better mental health. Although this questionnaire does not have a formal cut-off (Kelly et al., 2008), based on the most used one in the literature (Holmes, 1998; Thorsen et al., 2013), mental troubles were assessed with a score of 52 or less. Finally, we assessed the participants’ body image and trichotomised the answers into too thin (too thin/a little bit too thin), good, too fat (a little bit too fat/too fat).

### Data Analyses

The two groups (Low, High) were compared on the above-mentioned variables. For the bivariate analyses, we used chi-square tests for categorical variables and student t-tests for continuous ones to identify the characteristics associated with having low sexual desire. Statistically significant variables at the bivariate level were entered into a logistic regression analysis using the Low group as the reference category. Given that sample size was relatively large, we fixed the significance level of 1% to avoid Type-1 error. However, for the discussion and interpretation of the results at the multivariate level, we also considered the trend with the significance level of 5% (Barrense-Dias et al., 2020). All the analyses were performed separately by gender. To avoid missing data, we omitted individuals who did not complete the questionnaire to the end (Altman & Bland, 2007). For some questions (independent variables), participants could answer “other” or “I do not want to answer”. These few missing data (between 0.2 and 1.4%) did not reduce the statistical power of the analysis and were omitted in the analysis (Kang, 2013). Results are presented as Odds ratios (OR). We used STATA 16.0 (StataCorp, College Station, TX, USA) for all the analyses.

## Results

Overall, 11.3% of participants reported having a low sexual desire (Low group). The rates of females and males who had a low sexual desire were, respectively, 17.2% and 5.7%. At the bivariate level, a significant difference was found in terms of gender, with females being overrepresented in the Low group (data not shown).

### Females

#### Bivariate Analyses

*Sociodemographic Characteristics.* At the bivariate level, significant differences between the High and Low sexual desire groups were the fact of living with their parents and to be dissatisfied with their social life, with females in the Low group being overrepresented (Table 1).

**Table 1:** Sociodemographic data. Bivariate analysis comparing the two groups (Low and High) of sexual desire (females only)

	LOW (n = 431) % or mean	HIGH (n = 2072) % or mean	*p *value
Sociodemographic and personal data (n = 2503)			
Age (mean ± s.e.)	26.4 ± 0.04	26.3 ± 0.02	0.230
Swiss–born (yes)	88.4%	88.2%	0.924
Residence (urban)	51.5%	54.3%	0.258
Satisfaction with financial situation (of 1 to10), (mean ± s.e.)	6.3 ± 0.12	6.4 ± 0.05	0.171
Professional situation			0.3588
Job	68.7%	69.4%	
Student	19.5%	23.8%	
Other (housewife/husband, unemployed, disability/survivor’s insurance, social assistance, job search, other)	11.8%	6.8%	
Living with parents (yes)	24.7%	16.8%	** < 0.01**
Level of education (tertiary)	54.3%	55.6%	0.5986
Linguistic region (cantons)			0.3058
German	71.3%	68.1%	
French	25.8%	28.1%	
Italian	2.9%	3.8%	
Perceived puberty onset			0.3290
Advanced	29.0%	31.1%	
On time	41.0%	42.0%	
Delayed	30.1%	26.8%	

s.e., standard error

Chi–square tests and student* t-*tests for *p *value

Significant pvalues are in bold type

*Affective and sexual life.* Females in the Low group were less likely to consume pornography, to have current relationship(s) (casual or steady), to report a higher number of lifetime sexual partners and to be satisfied with their sexual life in the last 4 weeks (Table 2). No differences were found for sexual attraction.

**Table 2:** Affective and sexual life, unwanted sexual experiences and health data. Bivariate analysis comparing the two groups (Low and High) of sexual desire (females only)

	LOW % or mean	HIGH % or mean	*p *value
Affective and sexual life data			
Sexual attraction (non–hetero) (n = 2491*)	13.4% N = 430	10.6% N = 2060	0.0829
Pornography (several times) (n = 2503)	43.4% N = 431	50.3% N = 2072	**0.005**
Current relationship (n = 2496*)			** < 0.01**
None	37.3%	13.6%	
Occasional relation(s)—Steady relationship with occasional relations	3.9%	12.7%	
Steady relationship	58.8%	73.7%	
Number of lifetime sexual partners (n = 2485*)			** < 0.01**
0	13.3%	2.6%	
1	18.6%	15.4%	
2–3	19.2%	22.1%	
4 or +	48.9%	59.8%	
Satisfaction with sexual life in the last 4 weeks (very/somewhat dissatisfied) (n = 2468*)	27.2%	10.5%	** < 0.01**
Unwanted sexual experiences data			
Unwanted sexual intercourses or contacts (n = 2503)			0.1094
No	74.7%	75.1%	
Yes, once	14.6%	16.7%	
Yes, several times with the same person/various persons	10.8%	8.1%	
Ever accepted sexual intercourse without really wanting (n = 2503)			** < 0.01**
No	44.8%	47.5%	
Yes, once	15.2%	22.5%	
Yes, several times	40.0%	30.1%	
Ever been victim of sexual assault/abuse (n = 2503)	17.8%	15.6%	0.2219
General health data			
Health status perception (n = 2503)			
Excellent/very good	52.6%	61.8%	** < 0.01**
Good	38.6%	32.7%	
Mediocre/Poor	8.8%	5.4%	
Mental health (poor) (n = 2503)	24.2%	16.5%	** < 0.01**
Body perception (n = 2503)			**0.008**
Too thin/A little bit too thin	3.65%	3.1%	
Good	43.5%	51.3%	
Too fat/A little bit too fat	52.9%	45.6%	
Satisfaction with social life (of 1 to10), (mean ± s.e.) (n = 2503)	7.2 ± 0.1	8 ± 0.04	** < 0.01**

*Missing data: Some participants did not want to answer or answered other

s.e., standard error

Chi–square tests and student t-tests for *p* value

Significant pvalues are in bold type

*Undesired sexual experiences (USE). *Females in the Low group were more likely to have accepted several times sexual intercourse without really wanting (Table 2). No differences were found for unwanted sexual intercourse or contact and sexual abuse.

*Overall Health.* Females in the Low group were more likely to report both a poorer general and mental health status, and to perceive their body as too fat (Table 2 near here).

#### Multivariate Analyses

At the multivariate level (Table 3), compared to females in the High group, females in the Low group had lower odds of being satisfied with their social life (OR 0.89), having current relationship(s) (OR 0.16 for casual relationship(s); OR 0.54 for a steady relationship) and having had sexual partners in their life (OR 0.42 for one; OR 0.27 for two or three; 0.26 for four or more). They had higher odds of being dissatisfied with their sexual life in the past 4 weeks (OR 2.25) and having accepted several times sexual intercourse without really wanting (OR 2.01). There was also a trend regarding pornography use, with females in the Low group having lower odds of having watched pornography several times (OR 0.78).

**Table 3:** Multivariate logistic regression using HIGH as the Reference Category (females only)

	OR (95% CI) (n = 2443)
Living with parents (yes)	0.93 (0.7–1.24)
Pornography (yes)	0.78* (0.62–0.97)
Current relationship (none)	
Occasional relation(s)—Steady relationship with occasional relations	0.17** (0.09–0.30)
Steady relationship	0.54** (0.39–0.74)
Number of lifetime sexual partners (0)	
1	0.40** (0.23–0.71)
2–3	0.26** (0.15–0.45)
4 or +	0.25** (0.15–0.42)
Satisfaction with sexual life in the last 4 weeks (very/somewhat dissatisfied)	2.27** (1.63–3.16)
Ever accepted sexual intercourse without really wanting (no)	
Yes, once	0.98 (0.71–1.34)
Yes, several times	2.01** (1.56–2.59)
Health status perception (excellent/very good)	
Good	1.18 (0.93–1.50)
Mediocre/Poor	1.31 (0.82–2.08)
Mental health (poor)	0.99 (0.73–1.33)
Body perception (Too thin/A little bit too thin)	
Good	1.06 (0.56–1.98)
Too fat/A little bit too fat	1.18 (0.82–2.08)
Satisfaction with social life	0.89** (0.84–0.94)

Significant odds ratios (OR) are in bold type

Significant difference with the reference category (*p < 0.01) ** (*p* < 0.05)

*CI* confidence interval

### Males

#### Bivariate Analysis

*Sociodemographic Characteristics.* At the bivariate level, the only significant difference between the two groups was to be dissatisfied with their social life, with males in the Low group being more represented (Table 4).

**Table 4:** Sociodemographic characteristics. Bivariate analysis comparing the two groups (LOW and HIGH) of sexual desire (males only)

	LOW (n = 151) % or mean	HIGH (n = 2470) % or mean	*p *value
Sociodemographic and Personal Data (n = 2621)			
Age (mean ± s.e.)	26.4 ± 0.07	26.4 ± 0.02	NS 0.292
Swiss–born (yes)	85.4%	89.3%	0.1571
Residence (urban)	56.7%	52.2%	0.2369
Satisfaction with financial situation (of 1 to10), (mean ± s.e.)	6.4 ± 0.5	6.3 ± 0.1	0.768
Professional situation			0.2353
Job	64.3%	67.1%	
Student	22.3%	24.1%	
Other (housewife/husband, unemployed, disability/survivor’s insurance, social assistance, job search, other)	13.4%	8.8%	
Living with parents (yes)	35.3%	28.3%	0.098
Level of education (tertiary)	45%	46%	0.831
Linguistic region (cantons)			0.116
German	77.1%	68.5%	
French	21.2%	27.8%	
Italian	1.76%	3.8%	
Perceived puberty onset			0.209
Advanced	29.4%	22.5%	
On time	42.4%	47.9%	
Delayed	28.2%	29.6%	

Chi–square tests and student t tests for *p* value

Significant *p* values are in bold type

s.e., standard error

*Affective and sexual life.* Males reporting low sexual desire were more likely to report a non-heterosexual attraction, to consume less pornography, to have no current relationship (casual or steady), to report a lower number of lifetime sexual partners and to be dissatisfied with their sexual life in the last 4 weeks (Table 5).

**Table 5:** Affective and sexual life, unwanted sexual experiences and health data. Bivariate analysis comparing the two groups (Low and High) of sexual desire (males only)

Affective and sexual life data			
Sexual attraction (non–hetero) (n = 2600*)	18.3%	7.7%	** < 0.01**
Pornography (several times) (n = 2621)	81.9%	93.2%	** < 0.01**
Current relationship (n = 2617*)			** < 0.01**
None	60.6%	23.2%	
Occasional relation(s)—Steady relationship with occasional relations	11.6%	14.4%	
Steady relationship	27.8%	62.4%	
Number of lifetime sexual partners (n = 2587*)			** < 0.01**
0	25.7%	4.8%	
1	14.7%	13.3%	
2–3	15.5%	20.4%	
4 or +	44.2%	61.5%	
Satisfaction with sexual life in the last 4 weeks (very/somewhat dissatisfied) (n = 2593*)	38%	20.3%	** < 0.01**
Unwanted sexual experiences data			
Unwanted sexual intercourses or contacts (n = 2621)			0.608
No	91.6%	92%	
Yes, once	4.6%	5.6%	
Yes, several times with the same person/various persons	3.8%	2.5%	
Ever accepted sexual intercourse without really wanting (n = 2621*)			** < 0.008**
No	71.1%	77.4%	
Yes, once	10%	12.5%	
Yes, several times	18.9%	10.2%	
Ever been victim of sexual assault/abuse (n = 2621*)	2.9%	2.8%	0.222
General Health Data			
Health status perception (n = 2621*)			
Excellent/very good	53.6%	66.4%	**0.005**
Good	36.5%	28.6%	
Mediocre/Poor	10.0%	5.0%	
Mental health (poor) (n = 2621*)	24.1%	12.4%	** < 0.01**
Body perception (n = 2621*)			0.794
Too thin/A little bit too thin	8.7%	9.6%	
Good	54.7%	54.7%	
Too fat/A little bit too fat	36.55%	36.55%	
Satisfaction with social life, (of 1 to10), (mean ± s.e.) (n = 2621*)	6.6 ± 0.2	7.6 ± 0.04	** < 0.01**

*Missing data: Some participants did not want to answer or answered other

Chi–square tests and student t-tests for *p *value

Significant *p* values are in bold type

s.e., standard error

*Undesired Sexual Experiences (USE). *Participants in the Low group were more likely to have accepted several times sexual intercourse without really wanting (Table 5). No differences were found for unwanted sexual intercourse or contact and sexual abuse.

*General Health.* Males in the Low group were more likely to report both a poorer general and mental health status (Table 5 near here). No differences were found in terms of body image.

#### Multivariate Analyses

In the multivariate analysis (Table 6 near here), compared to males in the High group, males in the Low group had lower odds of having a current steady relationship (OR 0.29), casual relationships (trend) (OR 0.44), having watched pornography several times in their life (OR 0.44) and having had several sexual partners in their life (OR 0.36 for 2–3; 0.31 for four or more). They had higher odds of having a non-heterosexual sexual attraction (OR 1.99) (trend) and having accepted several times sexual intercourse without really wanting (OR 3.49).

**Table 6:** Multivariate logistic regression using HIGH as the Reference Category (males only)

	OR (95% CI) (n = 2542)
Sexual attraction (non–hetero)	1.99* (1.11–3.59)
Pornography (yes)	0.44** (0.24–0.8)
Current relationship (none)	
Occasional relation(s)—Steady relationship with occasional relations	0.44* (0.21–0.95)
Steady relationship	0.29** (0.15–0.57)
Number of lifetime sexual partners (0)	
1	0.52 (0.25–1.10)
2–3	0.36** (0.17–0.75)
4 or +	0.31** (0.15–0.62)
Satisfaction with sexual life in the last 4 weeks (very/somewhat dissatisfied)	0.90 (0.49–1.63)
Ever accepted sexual intercourse without really wanting (no)	
Yes, once	1.36 (0.67–2.78)
Yes, several times	3.49** (1.97–6.17)
Health status perception (excellent/very good)	
Good	1.46 (0.95–2.26)
Mediocre/Poor	1.22 (0.55–2.69)
Mental health (poor)	0.84 (0.5–1.41)
Satisfaction with social life	0.94 (0.85–1.04)

Significant odds ratios (OR) are in bold type

Significant difference with the reference category (*p < 0.01.) ** (*p* < 0.05)

*CI* confidence interval

## Discussion

### Sexual Desire (Overall Results)

As emphasized by literature (Dawson & Chivers, 2014; Dosch et al., 2016b), our results show that females have a higher probability than males to experience a low sexual desire with a prevalence of, respectively 17.2% and 5.7%. However, this study shows that males also report low sexual desire. Regarding females, our results are between those from a German study (Burghardt et al., 2020) that reported 12.6% of the 18–30 year-old females having an absent sexual desire and those from a Swiss study (Dosch et al., 2016b) that reported 26.5% of the 25–46 year-old females having a low level of sexual desire. Regarding males, our results are above those from another German study (Beutel et al., 2018) that reported 2.8% of the 18–30 year-old males having an absent sexual desire and those from the Swiss study (Dosch et al., 2016b) that found that all 25–46 year-old males had sexual desire. However, these two studies used a narrower definition of sexual desire (Beutel et al., 2018), a smaller sample and a broader age range (Dosch et al., 2016b).

Regarding the gender differences in terms of level of sexual desire, in addition to the diversity of factors which can influence females’ and males’ sexual desire in different ways, it is possible that social desirability led males reporting a higher level of sexual desire, and females, a lower level of sexual desire (Dawson & Chivers, 2014). Indeed, according to the double standard hypothesis concept, males and females are not judged the same way for similar sexual behaviors and the prevalent idea is that males often, if not always, have high sexual desire. (Dawson & Chivers, 2014).

### Affective and Sexual Life

First, females and males reporting a low sexual desire were more likely to have no current relation. These results could be explained by the fact that sexual desire may be increased with the desire of intimacy or closeness with a partner (Dawson & Chivers, 2014). Alternatively, having no desire may imply that an individual does not want to have a sexual partner. Among females, those who were in a steady relationship had higher odds of not having sexual desire than those who had casual relationships. This phenomenon had already been raised in a study (Bell et al., 2022) among young Norwegian adults females in which more females with a partner reported a lack of sexual desire than females without a partner. Among males, we did not find this trend. An explanation may be that males underreport a potential decrease of sexual desire contrary to females (Murray & Milhausen, 2012). Then, we may hypothesize that among the participants who reported a steady relationship, some of them where in a long-term relationship. The association between a relationship duration and the decline of sexual desire among females, but not among males, had already been raised in other studies (Mark & Lasslo, 2018; Murray & Milhausen, 2012). Some contextual factors due to the relationship length may have an impact on the sexual desire of females, such as a greater burden of responsibilities over time (Mark & Lasslo, 2018) but more research is needed on this topic.

Among both genders, those who reported a low sexual desire were more likely to have had no lifetime sexual partners. On the one hand, having many partners may be a consequence of having a high sexual desire (Dawson & Chivers, 2014). On the other hand, a study led in Switzerland (Ammar et al., 2014) found that having had few or no intimate partners was correlated with a low sexual desire. An explanation may be that sexual desire drives sexual behavior and therefore, the higher the level of sexual desire, the higher the number of sexual partners. Finally, among females, those who reported a low sexual desire were more likely to be dissatisfied with their sexual life. Although causality cannot be assessed here, it is possible that sexual dissatisfaction leads to a reduced level of sexual desire on the one hand. On the other hand, having a low sexual desire might lead to some sexual frustration and therefore, sexual dissatisfaction. It is however important to underline that one can have a low sexual desire and be sexually satisfied (Dosch et al., 2016b).

### Pornography

We found an association (a trend among females) between reporting a low sexual desire and not consuming pornography. Debates persist over the associations between pornography use and sexual desire. According to some authors (Krejčová et al., 2017; Willoughby & Leonhardt, 2020), pornography use is positively correlated with sexual desire, while others (Carvalheira et al., 2015; Dwulit & Rzymski, 2019; Pizzol et al., 2016) found that pornography use decreases sexual desire. People with no sexual desire may be less prone to watch pornography because they have a low sex drive and/or are not interested by things of sexual nature.

### Sexual Attraction

Among males, we found a trend between reporting a non-heterosexual sexual attraction and a low sexual desire. In line with these results, a study led online on various countries across the world (Lippa, 2007) and another one led in the United States (Welling et al., 2013) found that males with a heterosexual attraction had more sexual desire that the ones with a homosexual or bisexual one. According to some authors (Nimbi et al., 2020b), stereotypes and stigmatization may have an impact on the sexual desire of males who do not have an heterosexual attraction. The heteronormative standards may also lead them to underreport that they have sexual desire. This shows us that sexual desire encompasses social aspects and that it is important to analyze how stereotypes and social norms may influence our sexuality (Nimbi et al., 2020c). Finally, based on the same survey, Auderset et al. (2021) found that having a non-heterosexual attraction was, only among males, associated with having accepted sexual intercourse several times without really wanting. In our study, we found that this form of USE was associated with a low sexual desire.

### Undesired Sexual Experiences (USE)

Having accepted sexual intercourse several times without really wanting was associated with a low sexual desire among both females and males. In a study (Turchik & Hassija, 2014) led among 18–22 year-old females in the USA, a positive correlation was found between the lack of sexual desire and USE among females. Among 18–22 year-old males, contrary to our findings, USE was associated with a lack of sexual desire at the bivariate level, but not at the multivariate one (Turchik, 2012). Although causality cannot be assessed, on the one hand, sexual dysfunctions, which include sexual desire, are frequently reported by victims of undesired sexual experiences (McCool-Myers et al., 2018). On the other hand, people with a low sexual desire would tend to accept sexual intercourse to satisfy the partners’ expectations and do not dare say “no” for instance. Indeed, “to please”, “do not dare to say no” and “partner expected to have sexual intercourse” were among the most cited reasons given by both females and males in the main results of the basic survey (Barrense-Dias et al., 2018) for having accepted sexual intercourse without really wanting it. It is noteworthy that males who had accepted intercourse several times without really wanting it had more odds of reporting a low sexual desire than females who had accepted intercourse several times without really wanting it. These considerations show us the importance of promoting a culture of consent from an early age. Children need to learn how to set out their own boundaries and how to respect those of others.

## Strengths and Limitations

The first strength of this study was the sample size. Even though the response rate was low (15.1%) for the overall study, it was still a very large sample of this population on such a topic. Second, unlike most studies on sexual desire, we were able to take into account the youth perspective. Third, we studied it from a gender prism.

However, some limitations need to be put forward. First, the response rate was lower than expected even though this rate is similar to other studies on sexual behavior (Döring & Mohseni, 2018; Jørgensen et al., 2015). The fact that sexual health and behaviors are sensitive issues and that potential participants may not be at ease answering through the web (even if it was secured) could be an explanation. For these reasons we decided to start with a very large audience so that the final sample would be large enough for statistical purposes. Moreover, we could only contact participants through postal mail and having to connect to the website and introduce a code might have reduced the likelihood of answering compared to having received the invitation electronically. Additionally, the survey was conducted between June and September which coincided with the summer holidays in Switzerland. Second, considering that the survey was published online later than planned, the participants ended up being older than expected. Third, this was a cross-sectional study and no causality can be inferred. Fourth, regarding sexual desire we had to dichotomise the answers into two categories because of the small proportion of participants who had very low to low and no sexual desire. Moreover, we did not have separate data on respectively dyadic and solitary sexual desire. Fifth, as we did not have data on sexual desire before the last 4 weeks, we could not study the decrease or increase of sexual desire on a longer term basis. Finally, on the topic of pornography, we did not have data on the frequency of pornography consumption. Seventh, we could not analyze if there was an association between religious status and sexual desire as we did not ask participants if they were religious.

## Conclusion

These results show that sexual desire encompasses social aspects and underline the importance of addressing how stereotypes and social norms may influence our sexuality. They also demonstrate that sexual desire and sexuality both address an equal number of aspects: physical, social, mental and emotional ones. They must therefore be studied from a global point of view.

The prevailing idea that young people, particularly males, always have high levels of sexual desire may not be accurate and warrants further consideration. Researchers and clinicians should not overlook the fact that this population also has low levels of sexual desire and as a consequence the health staff should address how stereotypes and social norms may influence someone’s sexuality. It is important acknowledge that gender norms and the pressure put on males regarding sexual desire and activity may lead them to underreport a lack of sexual desire.

We may wonder if there really is a decrease in sexual desire amongst the youth as suggests (1, 3) or rather if individuals ask themselves more about whether or not they have sexual desire and/or dare to say more than before when they do not have desire. Some movements (e.g. #Metoo) indeed lead to the progress of social norms and the empowerment of females which could make easier for them to say they do not have sexual desire. The norms of masculinity, including the idea of a high libido, are also gradually deconstructed and may encourage more males to report a low level of sexual desire.

Sexual desire must be addressed in sexual education. The World Health Organization underlines the importance of talking about it in its Standards for Sexuality Education in Europe (50), stating that exploring sexual desires helps youth to learn about sexuality.

## Footnotes

1 Ethics Committee in research of the canton of Vaud
